# Structure–Property Relationships in Gamma-Irradiated Films Based on Gelatin/Modified Cassava Starch and Gelatin/Gluten Blends

**DOI:** 10.3390/polym18111337

**Published:** 2026-05-28

**Authors:** Larissa Canhadas Bertan, Farayde Matta Fakhouri, Gislaine Ferreira Nogueira, Daniela de Almeida Carrea, Marta Hiromi Taniwaki, Marina Venturini Copetti, Beatriz Thie Iamanaka, José Ignacio Velasco

**Affiliations:** 1 Faculty of Food Engineering, Federal University of Fronteira Sul (UFFS), Rodovia BR 158, Km 405, Laranjeiras do Sul 85319-899, PR, Brazil; larissa.bertan@uffs.edu.br; 2 Poly2 Group, Department of Materials Science and Engineering, Universitat Politècnica de Catalunya (UPC Barcelona Tech), 08222 Terrassa, Spain; daniela.de.almeida.carrea@upc.edu; 3 Department of Biomedical and Health Sciences, Minas Gerais State University, Passos 37900-106, MG, Brazil; gislaine.nogueira@uemg.br; 4 Food Science and Quality Center CCQA, Food Technology Institute ITAL, Av. 2880-Vila Nova, Campinas 13070-178, SP, Brazil; marta@ital.sp.gov.br (M.H.T.); beatriz@ital.sp.gov.br (B.T.I.); 5 Department of Food Science and Technology, Federal University of Santa Maria, Av. Roraima, n° 1000, Cidade Universitária, Bairro Camobi, Santa Maria 97105-900, RS, Brazil; marina.copetti@ufsm.br

**Keywords:** gamma irradiation, starch–protein composites, crosslinking, mechanical properties, barrier properties, antimicrobial activity

## Abstract

This study investigates the effect of gamma irradiation on the physicochemical, mechanical, and antimicrobial properties of biodegradable composite films based on modified cassava starch/gelatin (GS) and wheat gluten/gelatin (GG), including their active formulations with 2% potassium sorbate (GS2S and GG2S, respectively). Films were produced by casting and irradiated at 2–32 kGy. Irradiation modulated the structure–property relationships of starch–protein matrices in a dose-dependent manner. In GG systems, tensile strength increased while elongation decreased, indicating enhanced intermolecular interactions. At higher doses (16–32 kGy), excessive rigidity was observed, whereas lower doses (2–8 kGy) provided a more favorable balance between strength and flexibility. Water vapor permeability decreased in selected formulations, while solubility results indicated the coexistence of crosslinking and chain scission mechanisms. Among the tested conditions, 2 kGy was identified as the optimal dose. Despite the incorporation of 2% potassium sorbate, antimicrobial performance remained limited, with modest inhibition against *Wallemia sebi* and negligible effects on *Aspergillus chevalieri* and *Aspergillus montevidensis*. HPLC analysis demonstrated a significant reduction in sorbate release after irradiation (up to ~80%), indicating that restricted mass transfer, rather than intrinsic antimicrobial inefficiency, governs the observed behavior. The main novelty of this work lies in demonstrating that irradiation-induced structural modifications improve mechanical and barrier properties but simultaneously hinder active compound release. This decoupling between structural performance and functional activity highlights a critical limitation in irradiated active films. Overall, gamma irradiation at 2 kGy is effective for tuning material properties; however, controlling release kinetics is essential to achieve functional antimicrobial performance in biodegradable active packaging systems.

## 1. Introduction

The growing environmental impact associated with petroleum-based plastics has intensified global efforts toward the development of biodegradable and sustainable packaging materials [1]. Conventional synthetic polymers, widely used in food packaging due to their excellent mechanical and barrier properties, generate serious environmental concerns due to their persistence and accumulation after disposal [2]. At the same time, consumers increasingly demand minimally processed foods with extended shelf life, improved safety, and reduced environmental footprint. These combined factors have driven the search for biodegradable materials capable of partially or completely replacing synthetic packaging systems [3,4].

Edible and biodegradable films have emerged as promising alternatives due to their ability to act as semi-permeable barriers to moisture, oxygen, and carbon dioxide transfer [5,6]. In addition to their passive protective function, these materials may serve as carriers of active compounds, such as antimicrobial and antioxidant agents, enabling the development of active packaging systems. Active films can interact with the packaged food, reducing microbial growth and extending shelf life while maintaining sensory and nutritional quality [7,8]. However, most protein- and polysaccharide-based films are inherently hydrophilic, which limits their moisture barrier performance and represents one of the main technological challenges for their commercial application.

Among protein-based materials, gelatin has been widely investigated due to its excellent film-forming capacity, availability, relatively low cost, and favorable mechanical and optical properties [9,10]. Gelatin films are generally transparent and flexible, exhibiting good tensile strength [11]. Previous studies have demonstrated that structural modifications, including chemical crosslinking, enzymatic treatment, and physical methods, can improve mechanical resistance and reduce moisture permeability by promoting intermolecular interactions within the polymeric matrix [12,13,14,15].

Wheat gluten, another protein extensively studied for biodegradable film production, is characterized by its viscoelastic behavior and strong cohesive properties, which favor film formation [16]. Gluten is primarily composed of gliadin and glutenin fractions. The formation of a three-dimensional network during film development is largely governed by intermolecular interactions, particularly disulfide bonds, hydrogen bonding, and hydrophobic interactions [17]. Gluten-based films generally exhibit good mechanical strength; however, their barrier properties are strongly affected by relative humidity due to their hydrophilic character. Processing parameters such as pH, plasticizer type and concentration, protein content, and thermal treatment significantly influence the final structural organization and performance of gluten films [18,19].

Starch, a naturally abundant polysaccharide found in cereals, roots, and tubers, is considered one of the most promising raw materials for biodegradable film production due to its renewability, low cost, and capacity to form continuous matrices [20]. Starch consists mainly of amylose, a mostly linear molecule responsible for film formation, and amylopectin, a highly branched macromolecule [21]. Upon gelatinization in excess water, starch granules swell and lose their crystalline structure, allowing amylose chains to realign during drying and form hydrogen-bonded networks that generate cohesive films [22]. Although starch films can exhibit good mechanical resistance and oxygen barrier properties, they are highly sensitive to moisture and plasticization by water, which negatively affects their stability under high humidity conditions [23,24].

To overcome the limitations related to the mechanical performance and barrier properties of hydrophilic biopolymers, several modification strategies have been explored, including the incorporation of lipids, plant extracts, nanocomposites, chemical and enzymatic crosslinking, and other physical treatments [25,26,27,28,29].

Among these, gamma irradiation has gained attention as an effective physical method capable of inducing intermolecular crosslinking within polymeric matrices [30,31]. Gamma irradiation promotes the formation of free radicals that may lead to crosslinking or chain scission, depending on the polymer structure and irradiation conditions [32]. In protein- and starch-based films, controlled irradiation has been associated with increased tensile strength, reduced water vapor permeability, and improved structural cohesion [33]. According to Kyungkim et al. [32], irradiation in starch-based films improves functional properties, including mechanical performance and barrier characteristics, while reducing water vapor permeability. In addition, structural modifications at the granular level have also been reported, such as the formation of surface fissures and changes in crystalline organization, which can influence the thermal and physicochemical behavior of starch [34].

The incorporation of antimicrobial agents into film matrices has enabled the development of active packaging systems [8]. Antimicrobial films can reduce microbial growth by controlled migration of inhibitory compounds to the food surface, thereby extending the lag phase of target microorganisms and increasing a product’s shelf life [35,36]. The combination of antimicrobial incorporation and gamma irradiation may produce a synergistic effect, enhancing both structural integrity and microbial control, as demonstrated in edible coatings combined with low-dose γ-irradiation for shelf-life extension of food products [37].

Bakery products such as sliced bread are particularly susceptible to fungal contamination and moisture-related quality deterioration. Therefore, the development of biodegradable active films with improved mechanical and barrier properties represents a promising strategy for extending bread’s shelf life while reducing reliance on synthetic packaging materials [38].

Although the effects of gamma irradiation on biopolymer-based films have been previously reported, most studies have focused mainly on isolated changes in mechanical, barrier, or structural properties. In this context, the novelty of the present study lies in the integrated evaluation of irradiated active films based on starch–protein composite systems, considering not only structure–property relationships but also the release behavior and antimicrobial performance of potassium sorbate. This approach makes it possible to understand whether irradiation-induced modifications in the polymer matrix improve film properties without compromising the availability and functionality of the active compound.

Based on these considerations, gelatin, wheat gluten, and starch were selected as raw materials for the development of irradiated active films because of their distinct film-forming properties and technological advantages. Gelatin was chosen due to its ability to form resistant and transparent films, whereas wheat gluten was selected because it can produce flexible films. Starch, in turn, was included because it is a low-cost polysaccharide, widely available in nature, and known for its good oxygen barrier properties. The use of these three biopolymers allowed the comparison of protein-based, polysaccharide-based, and blended matrices, making it possible to investigate how differences in polymer composition affect film properties, antimicrobial performance, and the response to gamma irradiation. Therefore, the objective of this study was to develop and characterize irradiated active biofilms based on these materials, used individually and in combination. The films were evaluated in terms of mechanical, barrier, structural, and antimicrobial properties, with particular emphasis on understanding how gamma irradiation influence’s structure–property relationships and the release behavior of the incorporated active compound. In addition, the films were applied in the packaging of sliced bread to assess their performance under real storage conditions and to evaluate the relationship between material properties and functional effectiveness in a food system.

## 2. Materials and Methods

### 2.1. Materials

Raw materials used for film preparation were wheat gluten (Roquette, Passo Fundo, Rio Grande do Sul, Brazil), type A gelatin (Leiner Davis Gelatin, Amparo, Sao Paulo, Brazil), modified cassava starch (Amidomax 5500, Corn Products, Mogi Guaçu, Sao Paulo, Brazil), potassium sorbate (Chemco, Paramount, CA, USA) as antimicrobial agent, glycerol (Synth, Diadema, Sao Paulo, Brazil) as plasticizer, and glacial acetic acid (Synth, Diadema, Sao Paulo, Brazil) for pH adjustment.

### 2.2. Film Preparation

The film-forming solutions of gelatin, wheat gluten, and modified cassava starch were prepared separately before being mixed to obtain the composite films.

Gelatin film-forming solution was prepared by hydrating 10 g gelatin in 100 mL distilled water for 1 h at 25 °C, followed by solubilization at 55 °C for 10 min. Glycerol was added at 5% (*w*/*w*, dry gelatin basis).

Wheat gluten solution was prepared using gluten (5.0 g/100 mL), ethanol (32.5 mL/100 mL), distilled water (67.5 mL/100 mL) and acetic acid to adjust pH to 5. Glycerol was added at 20% (*w*/*w*, dry gluten basis). The mixture was stirred while heating to 70 °C and held for 5 min, then centrifuged at 5000 rpm for 20 min at room temperature to remove insoluble fractions [17].

Modified cassava starch solution was prepared using starch at 2% (*w*/*w*, dry basis) in 100 mL water with glycerol at 10% (*w*/*w*, dry starch basis), under stirring and heating in a water bath at 75 °C for 15 min.

Composite films were prepared by mixing the previously prepared film-forming solutions of wheat gluten/gelatin or modified cassava starch/gelatin at a ratio of 1:4 (*v*/*v*), corresponding to one volume of gluten or starch solution and four volumes of gelatin solution. This ratio was adopted to obtain composite matrices with gelatin as the main film-forming component, while incorporating gluten or starch to modify the structural, mechanical, and barrier properties of the films. The resulting film-forming solutions were cast onto Plexiglas plates using a fixed volume of 20 mL per plate and dried at room temperature for 24 h [12,39]. Potassium sorbate was incorporated into the composite film-forming solutions at 2% (*w*/*v*) to obtain active films. This concentration was selected based on previous studies and preliminary tests, aiming to provide sufficient antimicrobial activity while maintaining film integrity, homogeneity, and processability. A single potassium sorbate concentration was used because the main objective of this study was to evaluate the effects of polymer matrix composition and gamma irradiation on the performance of active films containing potassium sorbate.

### 2.3. Gamma Irradiation

Film-forming solutions and dried active films were exposed to gamma irradiation using cobalt as the radiation source at doses of 2, 4, 8, 16, and 32 kGy [40]. Prior to irradiation, dried films were conditioned at 25 °C and 52% relative humidity for 48 h. Irradiation was performed at Companhia Brasileira de Esterilização (Jarinu, SP, Brazil).

### 2.4. Film Characterization

Films were conditioned at 25 °C and 52% RH for 48 h before testing.

#### 2.4.1. Water Vapor Permeability (WVP)

WVP was measured gravimetrically at 25 °C following ASTM E96 [41]. Film disks (9.0 cm diameter) were sealed with paraffin onto aluminum permeation cells containing calcium chloride and placed in desiccators with saturated NaCl solution (75% RH). Mass gain was recorded every 24 h for 7 days (triplicate). The WVP was then calculated according to Equation (1). Results are reported as g·mm/(m^2^·day·kPa).

WVP = (S × e)/(A × ΔP) × 24(1)

#### 2.4.2. Water Solubility

Water solubility was determined according to Gontard et al. [16]. Film disks (2 cm diameter) were dried at 105 °C for 24 h to obtain initial dry mass, immersed in 50 mL distilled water under gentle agitation for 24 h, then re-dried at 105 °C for 24 h to obtain final dry mass. The moisture content was then calculated using Equation (2). The water solubility (WS) was calculated using Equation (3).

(2) $$\mathrm{Moisture}\;(\%)=\frac{{m_{1}-m}_{2}}{m_{1}}\times 100$$

where $m_{1}$ is the initial sample mass before drying and $m_{2}$ is the sample mass after drying.

(3) $$\mathrm{WS}\;(\%)=\frac{{m_{2}-m}_{s}}{m_{2}}\times 100$$

where $m_{2}$ is mass after drying and $m_{2}$ is the sample mass after solubilization.

#### 2.4.3. Mechanical Properties

Tensile strength and elongation at break were determined using a TA.XT2 texture analyzer (Stable Micro Systems, Surrey, UK), following ASTM D882-83 [42], at 25 °C. The initial grip separation was 50 mm, and the crosshead speed was 1 mm/s. Film specimens were cut into rectangular strips measuring 100 mm in length × 25 mm in width, and six replicates were tested for each formulation. Tensile strength (TS) and elongation at break (E) were calculated using Equations (4) and (5), respectively:

TS = F_m_/A (4)

where TS is the tensile strength (MPa), Fm is the maximum force at film rupture (N), and A is the cross-sectional area of the film specimen, calculated from the specimen width and film thickness (mm^2^).

E = d_r_/d_i_ × 100 (5)

where E is the elongation at break (%), d_r_ is the extension at the moment of rupture, corresponding to the difference between the grip separation at rupture and the initial grip separation, and d_i_ is the initial grip separation distance, corresponding to 50 mm.

#### 2.4.4. Antimicrobial Activity

Antimicrobial activity of the active films was evaluated using agar diffusion and colony-forming unit (CFU) assays. The fungal strains tested were *Aspergillus chevalieri*, *Aspergillus montevidensis*, and *Wallemia sebii*, cultivated on CY20S medium (Czapek Yeast Extract Agar supplemented with 20% sucrose).

Fungal strains were incubated at 25 °C for 7–15 days, depending on the species. After growth, spore suspensions were prepared, and their concentrations were determined by direct microscopic counting using a Petroff–Hausser chamber (Hausser Scientific, Horsham, PA, USA). The suspensions were adjusted to the following concentrations: *A. chevalieri* (6.0 × 10^6^ spores/mL; dilution 10^−2^), *A. montevidensis* (4.0 × 10^6^ spores/mL; dilution 10^−2^), *W. sebii* (1.4 × 10^6^ spores/mL; dilution 10^−1^), and *P. raistrickii* (1.0 × 10^6^ spores/mL; dilution 10^−2^).

Sterile Petri dishes containing solidified culture media were surface inoculated with 0.1 mL of the respective spore suspensions. Active film disks (2.5 cm in diameter), previously exposed to ultraviolet (UV) light for 2 min for surface sterilization, were aseptically placed onto the inoculated agar surface. Blank tests (films without inoculation) were also performed to verify potential contamination. Plates were incubated at 25 °C for 120 h.

For irradiated samples, films were previously exposed to gamma irradiation at 2 kGy and prepared by casting using a fixed volume of 20 mL, as defined during film development.

Antimicrobial effectiveness was assessed by measuring the presence and diameter of inhibition halos formed around the film disks [43]. Additionally, microbial growth was quantified by determining colony-forming units (CFU/mL), when possible. All experiments were performed in triplicate, and films without antimicrobial agents were used as controls.

#### 2.4.5. Morphology (SEM)

Surface and cross-section microstructure were examined by SEM (LEO 440i, Cambridge, UK) at 5 kV. Films were stored over silica gel at 25 °C for 7 days, fractured, mounted on aluminum stubs with carbon tape, gold-coated (92 Å) and observed at 1000× magnification.

#### 2.4.6. FTIR Spectroscopy

FTIR spectra were recorded using a Thermo Nicolet IR200 (Themo Fisher, Madison, WI, USA) with UATR (diamond crystal). Film spectra were collected from 4000 to 600 cm^−1^. Powder spectra (gluten, sorbate, starch) were collected in KBr (1%) from 4000 to 400 cm^−1^.

#### 2.4.7. Sorbate Release and Quantification (HPLC)

Film samples (12.5 cm^2^) were immersed in 4000 mL ultrapure water under stirring at 25 °C. Aliquots (20 μL) were withdrawn at time points from 30 to 3600. Potassium sorbate was quantified by HPLC (Shimadzu 10 AVP, Shimadzu Corporation, Kioto, Japón) using a Shim-pack ODS column (4.6 μm, 4.6 × 150 mm) with UV detection at 260 nm [44]. The mobile phase was acetonitrile/water/phosphoric acid 0.1% (60/39/1, *v*/*v*/*v*) at 1 mL·min^−1^.

### 2.5. Food Application

#### 2.5.1. Bread Production

Sliced breads were produced according to the formulation presented in Table A1. The ingredients were mixed for 5 min at low speed (900 rpm) and 8 min at high speed (1800 rpm). The dough was then divided into 530 g portions, allowed to rest for 10 min, molded, and subsequently fermented in a proofing chamber at 30 °C and 80% relative humidity for 2 h. After fermentation, the breads were baked at 180 °C for 35 min. The loaves were then cooled for 24 h, sliced, and packaged in polyethylene bags. Slicing was performed on the following day, after which the packaging experiments were conducted.

#### 2.5.2. Application of Biofilms for Sliced Bread Packaging

Active films based on gluten/gelatin (1:4) containing 2% potassium sorbate, irradiated with gamma radiation at 2 kGy, were used in the assays, while commercial low-density polyethylene (LDPE) packaging was used as control. For each treatment, three bread slices were used. Two packaging approaches were evaluated. In the first approach, bread slices were placed together, interleaved with the active films, and subsequently packed in LDPE bags, as described by Soares et al. [44].

The packaged samples were stored at controlled temperature (25 °C) for up to 10 days. Analyses were performed at defined intervals (days 1, 4, and 7). The evaluated parameters included weight loss and texture.

#### 2.5.3. Weight Loss

Weight loss was determined by recording the initial weight of the bread and subsequently weighing the product together with the film using a semi-analytical balance (Toledo PB 3002, Greifensee, Switzerland). Analyses were performed in quintuplicate.

#### 2.5.4. Instrumental Texture Analysis

Instrumental texture of the breads, defined as the maximum force required to compress two slices of bread, was determined using a TA.XT2 texture analyzer according to AACC Method 74-09 [45]. Results were expressed in gram-force (gf). The measurements were performed using a P/35R acrylic cylindrical probe operating in compression mode. The test speed was set at 1.7 mm s^−1^ with 40% compression of the sample, while the pre-test and post-test speeds were adjusted to 1.0 mm s^−1^ and 10.0 mm s^−1^, respectively. All analyses were carried out in nine replicates.

#### 2.5.5. Moisture

Moisture content of the bread samples was determined in triplicate by desiccation at room conditions for 24 h followed by oven drying at 130 °C until constant weight, according to AACC Method 44-15-02 [46].

#### 2.5.6. Water Activity

Water activity was determined using an AquaLab Cx 2T instrument (Decagon Devices Inc., Pullman, WA, USA) operating at 25 °C. Bread samples consisted of slices approximately 3 mm thick placed into sample capsules for measurement. Analyses were performed in triplicate.

#### 2.5.7. Microbiological Analysis

For microbiological analysis, 25 g of sliced bread were homogenized with 225 mL of 0.1% peptone water in a stomacher for 2 min. Serial decimal dilutions were then prepared, followed by spread plating in Dichloran 18% glycerol agar (DG18) for the enumeration of molds and yeasts and pour plating in Plate Count Agar (PCA) for the determination of total aerobic mesophilic microorganisms. The DG18 and PCA plates were incubated at 25 °C for 7 and 2 days, respectively. Microbial counts were expressed as colony-forming units per gram (CFU g^−1^) according to the methodology described by Downes and Ito [47]. All analyses were performed in triplicate.

#### 2.5.8. Extraction and Quantification of Potassium Sorbate

Potassium sorbate extraction from sliced bread was performed according to the method described by Silveira [43], with minor modifications. Two grams of previously ground bread were mixed with 40 mL of ethanol and agitated for 3 h at 90 rpm. Subsequently, the volume was adjusted to 100 mL with ultrapure water (18.2 MΩ). After an additional 15 min of agitation, the supernatant was filtered through a 0.22 μm membrane filter. Aliquots of 20 μL were used for potassium sorbate quantification by high-performance liquid chromatography (HPLC). The analysis was carried out using a Shimadzu chromatograph (Shimadzu Corporation, Kioto, Japón) (model 10 AVP) equipped with a Shim-pack ODS column (4.6 × 150 mm, 4.6 μm particle size) and a UV detector. The mobile phase consisted of 60% acetonitrile, 39% water, and 1% phosphoric acid (0.01%). The flow rate was maintained at 1 mL min^−1^, and detection was performed at 260 nm.

### 2.6. Statistical Analysis

Statistical analyses were performed using analysis of variance (ANOVA) with the software Statistica^®^ 5.0 (StatSoft Inc., Tulsa, OK, USA). Significant differences among means were determined using Tukey’s test at a significance level of *p* < 0.05.

## 3. Results and Discussion

### 3.1. Effect of Gamma Irradiation Dose on Barrier and Mechanical Properties

#### 3.1.1. Vapor Permeability and Water Solubility

Table 1 shows the effect of different gamma irradiation doses (2, 4, 8, 16, and 32 kGy) on the water vapor permeability and water solubility of the selected films.

For GS films, no significant differences (*p* ≤ 0.05) were observed at irradiation doses of 2, 4, and 8 kGy compared to the control. A reduction in WVP was only observed at higher doses (16 and 32 kGy). In the presence of 2% potassium sorbate, only the 4 kGy treatment resulted in reduced WVP relative to the control, although no significant differences were found when compared with the other doses (2, 8, and 16 kGy).

No statistically significant differences (*p* ≤ 0.05) were detected for GG films regardless of the irradiation dose. In contrast, GG2S exhibited a significant reduction in WVP at 2, 4, 8, and 16 kGy compared to the control, whereas no significant change was observed at 32 kGy.

These results are consistent with previous studies reporting that gamma irradiation can improve the barrier properties of polymeric systems by promoting structural rearrangements that reduce molecular mobility. For instance, de Lima et al. [48] observed a significant decrease in water vapor permeability in bacterial cellulose/kappa-carrageenan films after irradiation at 25 kGy, which was attributed to increased structural compactness and stronger intermolecular interactions, such as hydrogen bonding. Similarly, Novianto et al. [49] reported that gamma irradiation reduced the WVP of fish gelatin/agar films from 19.20 × 10^−3^ g mm cm^−2^ day^−1^ kPa^−1^ in the control to 14.89 × 10^−3^ g mm cm^−2^ day^−1^ kPa^−1^ at 30 kGy, due to the formation of a denser polymeric matrix. However, at 40 kGy, WVP increased again, indicating that excessive irradiation induced chain scission and created preferential pathways for water vapor diffusion. These findings support the hypothesis that moderate irradiation doses may improve barrier properties through structural reorganization, whereas excessive doses can promote degradation and compromise the integrity of the polymer network.

However, the absence of a consistent reduction in WVP at higher doses (e.g., 32 kGy) suggests that excessive irradiation may induce competing effects, such as chain scission, leading to increased free volume and potential disruption of the polymer network. This behavior indicates that the balance between crosslinking-like interactions and degradation processes plays a critical role in determining the final barrier performance of irradiated films.

Furthermore, the non-homogeneous behavior observed among formulations suggests that the irradiation effects were strongly dependent on film composition and structural organization. Moderate irradiation doses may have promoted partial matrix densification and improved intermolecular interactions, reducing molecular mobility and limiting water vapor diffusion. This interpretation is supported by SEM observations, which revealed compact and continuous morphologies without visible pores in most irradiated films, indicating preservation or slight reorganization of the polymer network. In contrast, at higher doses, the increase in water vapor permeability observed in some formulations may be associated with chain scission and disruption of intermolecular interactions, leading to increased free volume within the matrix. FTIR results corroborate this interpretation, since irradiation caused subtle changes in band intensity and hydrogen bonding interactions without major chemical modifications, suggesting that the effects occurred mainly at the molecular level. Differences among formulations may also be related to the distinct structural organization of starch- and gluten-based matrices and to the presence of potassium sorbate, which appeared to promote more homogeneous and compact structures in SEM micrographs. Therefore, the permeability behavior likely reflects the competition between structural stabilization and degradation processes induced by gamma irradiation.

Regarding water solubility, GS films showed reduced solubility at 2, 4, and 16 kGy compared to the non-irradiated control, with no significant differences among these doses. Conversely, solubility increased at 8 and 32 kGy. In GS films containing 2% sorbate, a significant increase in solubility was observed only at 16 kGy, whereas no statistically significant changes were detected at the other doses.

A different trend was observed for GG films, where only the 2 kGy dose led to a significant increase in solubility compared to the control, while higher doses showed no significant effect. The non-irradiated GG2S exhibited higher solubility than the irradiated samples, and no statistically significant differences were observed among the dose pairs 2–32, 2–16, and 4–16 kGy.

The reduction in solubility observed at specific irradiation doses can be associated with decreased water–polymer interactions and reduced availability of hydrophilic sites. According to Bansal and Arora [50], irradiation can decrease water uptake in polymeric composites by reducing amorphous regions and improving interfacial interactions, which limits the accessibility of water molecules within the matrix. Similarly, Novianto et al. [49] reported that the solubility of fish gelatin/agar films decreased from 41.34% in the control to 25.79% at 30 kGy, due to the formation of intra- and intermolecular interactions that restricted water penetration and reduced gelatin–water interactions. However, at 40 kGy, solubility slightly increased again, suggesting that chain scission and degradation processes became predominant over structural stabilization. Likewise, Sarmast et al. [51] described similar reductions in water solubility in gamma-irradiated gelatin-based films. Studies on polyethylene- and polypropylene-based composites have also shown that irradiation reduces hydroxyl group availability and restricts water penetration, resulting in lower absorption and swelling. On the other hand, the increase in solubility observed at higher doses may be related to chain scission and molecular weight reduction, which enhances the formation of smaller, more water-soluble fragments. This behavior is consistent with the findings of Krieghoff et al. [52], who reported that irradiation-induced degradation in biodegradable polymers leads to the formation of low-molecular-weight oligomers that are more readily soluble in aqueous environments. Therefore, the solubility behavior observed in this study reflects a competition between structural stabilization at moderate doses and degradation processes at higher irradiation levels.

Overall, the results indicate that gamma irradiation can modulate both barrier and water interaction properties of polymeric films, but its effects are highly dose-dependent. While moderate irradiation doses may enhance structural organization and reduce permeability and solubility, excessive doses can promote degradation, increasing solubility and compromising barrier performance. This balance between improved intermolecular interactions and chain scission is a key factor in determining the functional properties of irradiated biopolymer films.

#### 3.1.2. Mechanical Properties

For GS films, irradiation generally reduced tensile strength compared to the non-irradiated control, regardless of the applied dose (Table 2). No statistically significant differences were observed between 2 and 4 kGy, nor among the dose groups 4–8–16 kGy and 8–16–32 kGy. A similar trend was observed for GS2S, where no significant differences were found between 16 and 32 kGy, as well as between 2 and 8 kGy and 2 and 4 kGy.

This reduction in tensile strength suggests that chain scission may be the dominant mechanism in GS systems under gamma irradiation. Similar behavior has been reported for biodegradable and semi-crystalline polymers, in which irradiation induces a decrease in molecular weight and compromises mechanical integrity. According to Krieghoff et al. [52], irradiation of polymers such as PLGA leads to chain cleavage and reduced structural cohesion, which negatively affects macroscopic mechanical performance. This mechanism is consistent with the behavior observed in the present starch–gelatin systems, which are more susceptible to degradation due to their hydrophilic and less densely packed structure. Furthermore, Zhou et al. [53] reported that gamma irradiation may induce surface deformation and microcracks in starch granules, which can compromise the structural uniformity of starch-based materials. This effect may contribute to the reduction in tensile strength observed in GS films after irradiation.

In contrast, irradiation increased the tensile strength of GG and GG2S films at all tested doses compared to the control. This behavior is likely associated with the presence of sulfur-containing amino acids in gluten proteins, which are particularly sensitive to radiation-induced modifications [54]. The absorbed energy may promote structural rearrangements and the formation of intermolecular interactions, resulting in enhanced mechanical resistance.

This improvement in tensile strength at lower irradiation doses is consistent with literature reports indicating that irradiation can promote the formation of reactive sites and enhance intermolecular interactions within polymer matrices. Bansal and Arora [50] reported that polymers such as polypropylene (PP) and polyethylene (PE) exhibit increased tensile strength at moderate irradiation doses due to the formation of covalent bonds and improved chain interactions. In composite systems, this effect is even more pronounced, as irradiation enhances interfacial adhesion between components, leading to significant improvements in mechanical performance. Although the present systems are not fiber-reinforced composites, the protein–polymer interactions in gluten-based films may play a similar role, promoting network reinforcement under irradiation. Similar behavior was reported by Novianto et al. [49], who observed a significant increase in tensile strength of fish gelatin/agar films after gamma irradiation. Tensile strength increased from 50.59 MPa in the non-irradiated control to a maximum value of 62.87 MPa at 30 kGy, indicating reinforcement of the polymeric network and improved structural integrity. However, at 40 kGy, tensile strength decreased to 53.78 MPa, suggesting that excessive irradiation promoted degradation and chain scission, which compromised the mechanical resistance of the films. These findings reinforce the hypothesis that moderate irradiation doses favor structural stabilization, whereas excessive doses may negatively affect mechanical performance.

Among the tested conditions, the 2 kGy dose was the most effective in increasing tensile strength, leading to an improvement of approximately 100% compared to the control. No statistically significant differences were observed between the dose pairs 16–32 kGy and 4–8 kGy. A similar trend was observed for GG2S, where 2 kGy produced the greatest increase in tensile strength, while no significant differences were found among 2–4–8 kGy or between 16 and 32 kGy.

The existence of an optimal irradiation dose is widely reported in the literature. According to Bansal and Arora [50], tensile strength typically increases up to a certain irradiation level, after which degradation processes such as chain scission become predominant, leading to a decline or stabilization in mechanical properties. A similar trend was reported by Novianto et al. [49], who identified 30 kGy as the most effective dose for improving the functional properties of fish gelatin/agar films. At this dose, water vapor permeability and water solubility were minimized, while tensile strength reached its highest value. However, at 40 kGy, the films showed increased permeability and solubility, accompanied by reduced tensile strength, indicating that degradation effects became predominant. This explains the plateau observed at higher doses (16 and 32 kGy) in the present study, where additional irradiation did not result in further improvements in tensile strength.

The effect of irradiation on elongation at break is also presented in Table 3. For both GS and GG films, irradiation did not significantly affect elongation compared to the non-irradiated control at any of the evaluated doses. In contrast, films containing 2% sorbate (GG2S and GS2S) showed a reduction in elongation after irradiation, although no statistically significant differences were observed among the applied doses.

The reduction in elongation observed in sorbate-containing films indicates a decrease in flexibility, which may be associated with reduced chain mobility and increased stiffness of the polymer network. This behavior is consistent with previous studies on irradiated polyethylene and polypropylene systems, where elongation at break decreases progressively with increasing irradiation dose, leading to more brittle materials [50]. The presence of sorbate may further influence this behavior by acting as a plasticizer prior to irradiation, while post-irradiation structural rearrangements restrict molecular mobility, resulting in lower elongation capacity.

In contrast, Novianto et al. [49], reported that elongation at break remained relatively constant (approximately 3.6%) across all evaluated irradiation doses, suggesting that gamma irradiation strengthened the polymeric network without significantly compromising film flexibility. Differences between the present study and the literature may be associated with variations in film composition, particularly the presence of potassium sorbate and the distinct structural organization of starch- and gluten-based matrices.

Based on the results obtained for the different irradiation doses, the 2 kGy dose was selected for the subsequent stages of the study. This choice was based on the improvement of mechanical properties, with increased tensile strength in two of the four films evaluated, in addition to avoiding the high stiffness observed at the higher doses (16 and 32 kGy). Furthermore, irradiation at 2 kGy reduced water vapor permeability, a desirable characteristic for packaging applications. This selection is also supported by literature findings indicating that low irradiation doses are often sufficient to promote beneficial structural modifications without inducing excessive degradation. As reported by Bansal and Arora [50], moderate doses optimize the balance between crosslinking-like effects and chain scission, resulting in improved mechanical performance while maintaining material integrity. Therefore, the choice of 2 kGy represents a compromise between enhancing functional properties and preserving the structural stability of the films.

#### 3.1.3. Evaluation of Antimicrobial Activity by Agar Diffusion of Irradiated Films

The antimicrobial activity of the films was evaluated against different fungal strains using agar diffusion assays (Table 3). The results for *A chevalieri* (Table 4) showed that the incorporation of 2% potassium sorbate into GS irradiated and GG irradiated films did not result in statistically significant differences (*p* > 0.05) in colony-forming units (CFU) compared to the control films. This behavior may be attributed to limited diffusion of potassium sorbate within the polymeric matrix, since antimicrobial effectiveness in active films depends primarily on the release and migration of the compound to the surrounding medium [55]. In protein- and starch-based systems, strong intermolecular interactions can restrict molecular mobility, reducing the availability of the active agent at the interface.

This result contrasts with findings reported by de Lima et al. [48], in which gamma irradiation (25 kGy) enhanced antimicrobial activity in cellulose-based films, leading to measurable inhibition halos even in matrices that previously showed low activity. In that study, irradiation promoted increased diffusion and/or activation of bioactive compounds, suggesting that the effect of irradiation on antimicrobial performance is strongly dependent on both the polymer matrix and irradiation dose.

These structural and diffusional limitations may have been further intensified by gamma irradiation, which, as indicated by HPLC (Section 3.1.6) results, reduced the effective release of potassium sorbate from the films, thereby limiting its antimicrobial activity in diffusion-based assays. Additionally, irradiation (2 kGy) may have modified the polymer network through chain scission, reducing molecular weight and generating structural discontinuities. These changes can increase matrix hydrophilicity and create microvoids, potentially enhancing water uptake and affecting sorbate mobility [56]. However, in control films without sorbate, irradiation did not improve antimicrobial performance, indicating that structural changes alone were insufficient to promote effective diffusion. According to de Lima et al. [48] and Zaki et al. [57], gamma irradiation can generate free radicals capable of enhancing antimicrobial activity by increasing the reactivity of incorporated compounds or promoting the breakdown of larger molecules into more bioavailable fractions. However, in the present study, this potential benefit was not observed, likely due to restricted mobility of potassium sorbate within the dense polymer network, highlighting that radical formation alone is not sufficient without effective release mechanisms.

In addition to CFU quantification, the inhibition halo was measured as the shortest distance between the film and the nearest colony, due to the non-uniform distribution of microbial growth. The inhibition halo ranged from 0 to 1.66 ± 1.15 mm, with no statistically significant differences (*p* > 0.05) among treatments according to Tukey’s test. These results indicate limited antimicrobial effectiveness against *A. chevalieri* under the tested conditions. The absence of inhibition zones in several treatments reinforces that potassium sorbate action was mainly contact-dependent, which is consistent with systems where diffusion is slow or restricted by the matrix structure.

For *A. montevidensis* no significant differences (*p* ≤ 0.05) in CFU counts were observed between films with and without sorbate. Although ANOVA indicated a significant overall effect for inhibition zone, Tukey’s test did not detect significant pairwise differences among formulations (*p* > 0.05). The slight increase in inhibition halo with sorbate incorporation was not statistically significant. This discrepancy is likely due to high within-group variability and limited sample size, which are common in diffusion-controlled systems. Similar inconsistencies between statistical significance and observable antimicrobial trends have been reported in diffusion-based systems, where heterogeneous release profiles limit reproducibility [48]. Moreover, Jagtap et al. [58] demonstrated that irradiation-induced antimicrobial effects can be more pronounced in surface-driven mechanisms (e.g., biofilm inhibition) rather than diffusion-dependent assays, reinforcing the importance of the evaluation method.

The presence of potassium sorbate may also contribute to increased free volume within the polymer network by reducing intermolecular interactions and acting as a plasticizer. Free volume facilitates molecular mobility and diffusion. When combined with irradiation-induced chain scission, this effect may further loosen the polymer structure, slightly enhancing swelling and interfacial contact with the culture medium. Nevertheless, these effects were not sufficient to produce consistent statistical differences.

In contrast, for *Wallemia sebii*, the addition of 2% potassium sorbate did not result in consistent statistically significant reductions in CFU across all formulations when evaluated by Tukey’s test. Although numerical reductions in fungal growth were observed, particularly in sorbate-containing films, these differences were not always statistically significant (*p* > 0.05). Potassium sorbate is known to inhibit fungi by disrupting membrane transport and intracellular pH balance, being particularly effective against molds and yeasts under favorable conditions [59]. Therefore, the greater numerical sensitivity of *W. sebii* may be related to species-specific physiological differences.

This species-dependent response is consistent with the selective antimicrobial behavior reported by de Lima et al. [48], where irradiated films showed higher effectiveness against Gram-positive microorganisms compared to Gram-negative ones, indicating that microbial structure and physiology significantly influence susceptibility.

**Figure 1:**
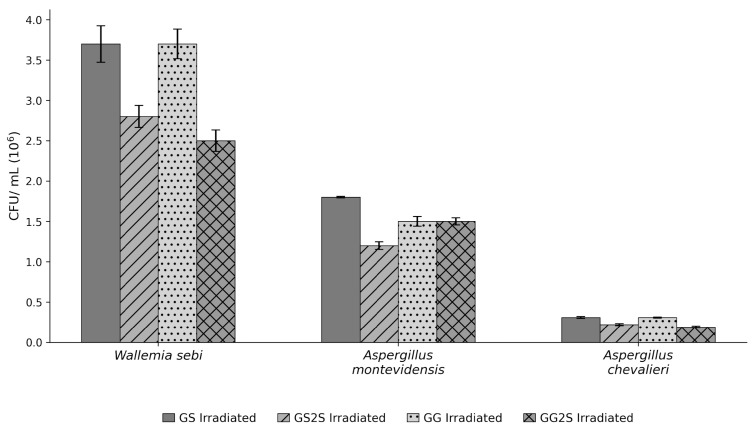
Fungal growth in colony forming units (CFU) of 3 different species: *Wallemia sebi*, *Aspergillus montevidensis*, *Aspergillus chevalieri* on different active films. GS = gelatin and starch; GG = gelatin and gluten; 2S = 2% potassium sorbate.

The GG formulation showed the most pronounced effect, with lower CFU values and larger inhibition halos (Figure 1); however, only the inhibition zone GG2S irradiated showed a tendency toward higher values, while other comparisons did not differ significantly. For GS2S irradiated, no statistically significant differences were observed between control and sorbate-containing films for inhibition halo. Differences between matrices can be explained by release kinetics: gelatin-rich systems tend to swell and release active compounds more slowly, whereas gluten-based films may facilitate greater sorbate migration under certain conditions [60].

Mass transport in these systems is governed by diffusivity and solubility. Diffusivity reflects molecular mobility within the matrix, while solubility describes the compatibility between sorbate and the polymer network. Factors such as pore size, chain flexibility, and polymer packing density, as well as environmental conditions (pH, water activity, and temperature), influence these parameters. Irradiation-induced structural changes may alter both diffusivity and solubility, resulting in heterogeneous and time-dependent release behavior. However, in the present study, this effect was not sufficient to produce consistent statistical separation.

In addition, irradiation-induced surface modifications, such as increased roughness and formation of nanostructures, have been reported to contribute to antimicrobial activity by limiting microbial adhesion and biofilm formation [58]. However, such mechanisms are more relevant in solid surface applications and may not significantly influence diffusion-based antimicrobial assays, as observed in the present study.

Overall, the antimicrobial performance of the films depended on both the fungal species and the film composition. While limited effects were observed for *Aspergillus chevalieri* and *Aspergillus montevidensis*, more pronounced inhibition was detected for *Wallemia sebi.* The GG2S formulation tended to show better antimicrobial performance compared to GS, particularly in terms of inhibition halo formation. Nevertheless, the overall antimicrobial response was constrained by diffusion-controlled release mechanisms, which are typically described by Fickian or quasi-Fickian behavior in biopolymer films [60]. This indicates that optimizing release kinetics is essential to enhance antimicrobial efficacy.

Overall, the present findings differ from studies employing higher irradiation doses (25–60 kGy), where significant antimicrobial enhancement has been reported [48,58]. This discrepancy reinforces that the antimicrobial effectiveness of irradiated films depends not only on the presence of active compounds but also on irradiation dose, matrix composition, and the balance between structural modification and controlled release.

Based on these results, GG2S and GS2S films were selected for subsequent experiments, due to their consistent trends in antimicrobial activity, even in the absence of statistically significant differences in all parameters. These formulations represent a compromise between mechanical performance and controlled release, which is a critical requirement for the development of effective antimicrobial packaging systems.

#### 3.1.4. Morphology

**Figure 2:**
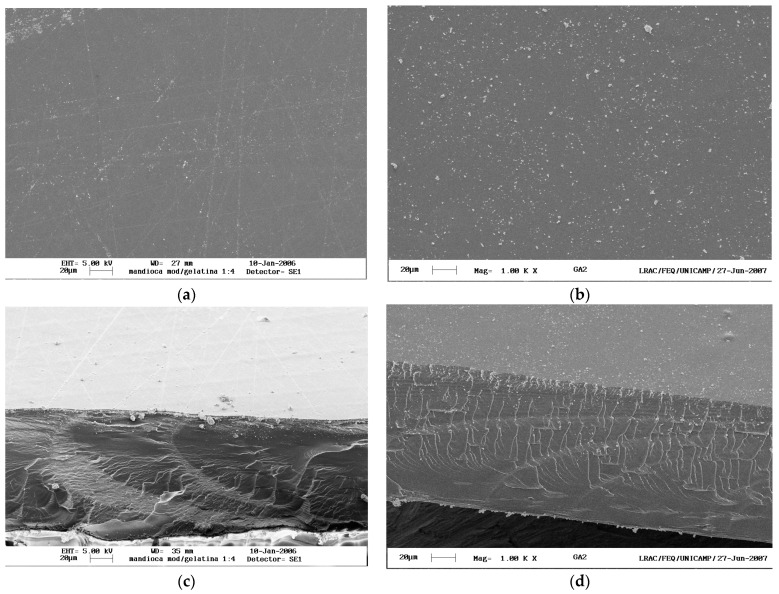
Micrographs obtained by SEM for GS films: (**a**) Surface of GS; (**b**) Surface of GS irradiated (2 kGy); (**c**) Cross-section of GS; (**d**) Cross-section of GS irradiated (2 kGy). GS = gelatin and starch.

**Figure 3:**
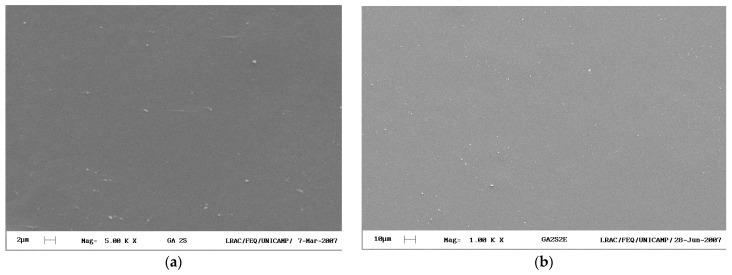

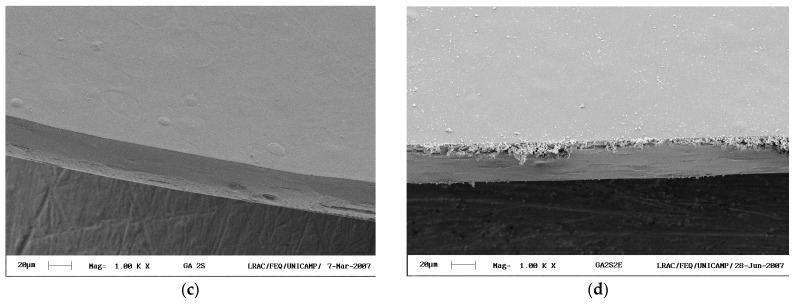
Micrographs obtained by SEM for GS2S films: (**a**) Surface of GS2S; (**b**) Surface of GS2S irradiated 2 kGy); (**c**) Cross-section of GS2S; (**d**) Cross-section of GS2S irradiated (2 kGy). GS = gelatin and starch; 2S = 2% potassium sorbate.

**Figure 4:**
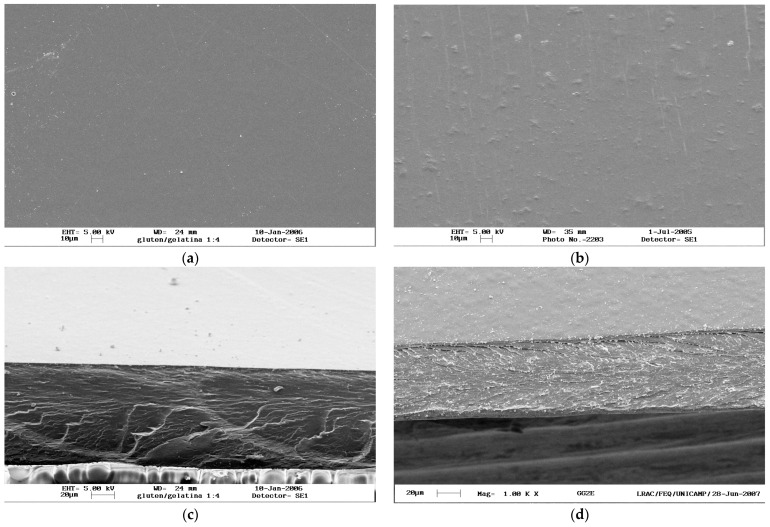
Micrographs obtained by SEM for the GG films: (**a**) Surface of GG; (**b**) Surface of GG irradiated (2 kGy); (**c**) Cross-section of GG; (**d**) Cross-section of GG irradiated (2 kGy). GG = gelatin and gluten.

**Figure 5:**
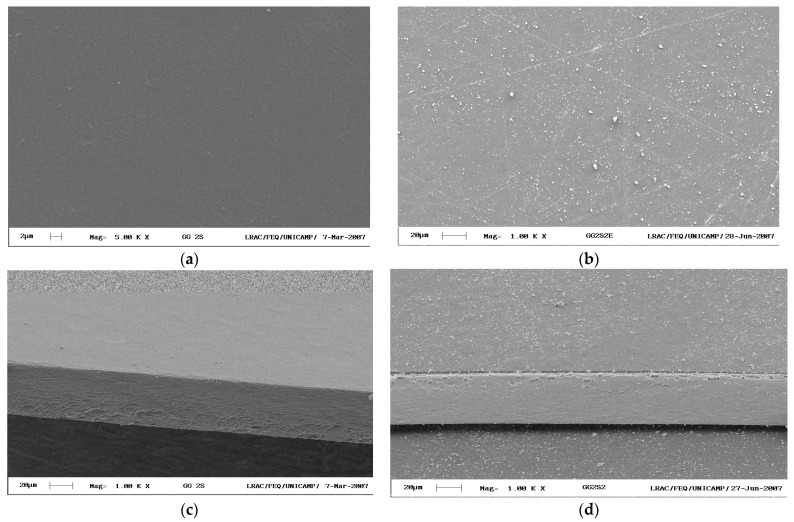
Micrographs obtained by SEM for the GG2S films: (**a**) Surface of GG2S; (**b**) Surface of GG2S irradiated (2 kGy); (**c**) Cross-section of GG2S; (**d**) Cross-section of GG2S irradiated (2 kGy). GG = gelatin and gluten; 2S = 2% potassium sorbate.

Scanning electron microscopy (SEM) micrographs of films prepared with and without potassium sorbate and subjected or not to gamma irradiation (2 kGy) are presented in Figure 2, Figure 3, Figure 4 and Figure 5. Surface and cross-sectional morphologies were evaluated to investigate possible structural changes induced by irradiation.

Overall, all film formulations exhibited continuous and compact morphologies, with no visible pores or major structural disruptions after irradiation. Non-irradiated films generally showed smooth and homogeneous surfaces, indicating good compatibility among the film-forming components and uniform matrix formation [58]. Similar morphologies were maintained after irradiation, suggesting that the applied dose (2 kGy) was not sufficient to promote pronounced microstructural damage.

Localized surface heterogeneity and small fragments were occasionally observed in irradiated samples, particularly in GS and GG films (Figure 2b and Figure 4b). However, these structures appeared superficially deposited and were also detected in fractured cross-sections, suggesting that they were mainly associated with sample fragmentation during SEM preparation rather than intrinsic morphological alterations. Since irradiation may increase film rigidity, fracture during stub preparation could contribute to the deposition of fragments on the film surface. Although irradiation-induced roughness and discontinuities have been reported as intrinsic radiation effects associated with chain scission and structural rearrangements [58,61], such effects were not predominant in the present study.

Cross-sectional images revealed compact internal structures, with fibrillar organization particularly evident in GS and GG films (Figure 2c,d and Figure 4c,d). This morphology is likely related to the entangled gelatin network and its triple-helix organization, which contributes to film structural integrity. Small white streaks observed within some matrices may be associated with glycerol phase separation. No substantial differences were detected between irradiated and non-irradiated samples in the cross-sectional morphology.

Films containing potassium sorbate (GS2S and GG2S; Figure 3 and Figure 5) exhibited particularly homogeneous and dense internal structures, suggesting improved compatibility between film components. The incorporation of low-molecular-weight compounds may contribute to matrix homogeneity by occupying free volume and modifying polymer–polymer interactions [50]. In these formulations, irradiation did not visibly alter either the surface or the internal morphology.

The preservation of compact and continuous morphologies after irradiation is consistent with previous reports indicating that moderate irradiation doses may promote subtle structural reorganization and increased intermolecular interactions without causing extensive morphological disruption [48]. More pronounced effects, such as crack formation, roughness, and nanostructuring, are generally associated with higher irradiation doses [58,61]. Similar behavior has been reported for irradiated pectin/gelatin and soy protein isolate films, in which irradiation improved matrix homogeneity and reduced structural discontinuities [62].

Overall, SEM observations indicate that gamma irradiation at 2 kGy did not induce major morphological changes in the polymer matrices. The films maintained structural integrity, suggesting that the irradiation effects observed in barrier and antimicrobial properties are more likely related to molecular-level modifications, such as rearrangement of intermolecular interactions, changes in free volume, and limited chain scission, rather than to large-scale microstructural alterations [63]. These observations are consistent with the FTIR results, which also indicated only subtle structural modifications after irradiation.

#### 3.1.5. FTIR Spectroscopy

**Figure 6:**
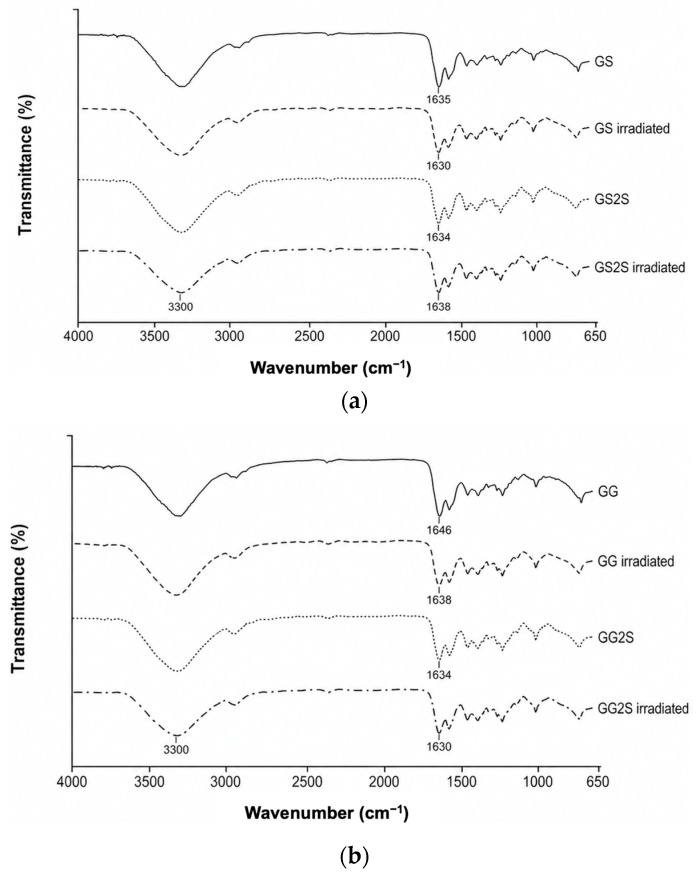
FTIR spectra of films (**a**) based on gelatin/starch formulations (GS and GS2S) and (**b**) based on gelatin/gluten formulations (GG and GG2S). GS = gelatin and starch; GG = gelatin and gluten; 2S = 2% potassium sorbate.

The main absorption bands were observed in the regions 1800–1000 cm^−1^ and 2900–3300 cm^−1^, typically associated with protein and polysaccharide structures present in the polymer matrix (Figure 6).

In GS films (Figure 6a), the disappearance of bands between 3900 and 3600 cm^−1^ and 1800–1700 cm^−1^ was observed after the addition of potassium sorbate. This behavior suggests modifications in the three-dimensional organization of the polymer network caused by the plasticizing effect of sorbate. The incorporation of low-molecular-weight compounds may reduce intermolecular interactions and increase chain mobility, affecting the vibrational modes detected in the spectra.

After gamma irradiation, the disappearance of these bands was also observed in GS and GS2S films, although no major changes in the overall spectral profile were detected. These effects may be associated with irradiation-induced structural rearrangements, such as crosslinking or chain scission, which can modify molecular conformation and vibrational intensities. Only vibrations that produce changes in the molecular dipole moment are detectable in infrared spectroscopy [64].

This behavior is consistent with previous studies reporting that gamma irradiation predominantly induces chain scission, leading to a reduction in the intensity of characteristic absorption bands rather than the formation of new functional groups [58,65]. For example, Chikaoui [65] observed a progressive decrease in FTIR band intensities in irradiated PET films, which was attributed to degradation of the polymer backbone. Similarly, Jagtap et al. [58] reported significant reductions and even disappearance of absorption bands in irradiated polycarbonate, indicating cleavage of C–O–C and carbonate linkages.

Several absorption bands between 1800 and 1000 cm^−1^ were mainly attributed to the gelatin component of the films. The intense bands observed between approximately 1650–1540 cm^−1^ correspond to the characteristic Amide I and Amide II bands, typical of protein structures. The Amide I band, mainly associated with C=O stretching vibrations of peptide bonds, is highly sensitive to protein secondary structure and provides information on conformational organization within the matrix. The Amide II band, attributed to N–H bending coupled with C–N stretching vibrations, reflects hydrogen bonding interactions within the gelatin network [64]. The presence of these bands indicates that the main protein structure of gelatin remained preserved after irradiation.

The preservation of these characteristic amide bands suggests that, at the applied dose (2 kGy), irradiation was not sufficient to cause extensive degradation of the protein backbone, which agrees with observations in biopolymer systems where moderate irradiation doses induce limited structural changes without complete disruption of the primary chemical structure [48].

A band around 1235 cm^−1^, corresponding to the Amide III region, is also identified in Figure 6a, associated with C–N stretching and N–H bending vibrations of peptide bonds. Additionally, a band near 1450 cm^−1^ was attributed to C–H vibrations of pyrrolidine rings present in proline and hydroxyproline residues characteristic of gelatin.

A broad band between 3060 and 3330 cm^−1^, observed in Figure 6a, was assigned to symmetric and asymmetric N–H stretching vibrations. In solid samples, these vibrations appear as broad bands due to extensive hydrogen bonding interactions between peptide groups [64]. Changes in this region after irradiation may also reflect modifications in hydrogen bonding interactions, as reported for PEO/PVA systems, where irradiation altered O–H stretching bands due to rearrangement of intermolecular interactions and formation of new radical sites [66,67].

In addition to protein-related bands, characteristic absorptions of cassava starch were detected in the region 1200–900 cm^−1^, corresponding to polysaccharide vibrational modes [53]. A band near 1020 cm^−1^ was observed and attributed to C–O and C–O–C stretching vibrations of glycosidic bonds in amylose and amylopectin. Similar bands around 1023 cm^−1^ have been reported for starch-based materials [54].

In irradiated polysaccharide-based systems, variations in this region are often associated with structural reorganization and increased amorphous character due to chain scission, as reported for polymer electrolytes and biopolymer composites [48,61]. The FTIR spectra of GG and GG2S films are presented in Figure 6b. Overall, films prepared with GG and GS showed very similar spectra regardless of irradiation (Figure 6). The main absorption bands in GG films were also observed in the regions 1800–1000 cm^−1^ and 2900–3300 cm^−1^, similarly to the GS.

As observed in starch-based films, intense bands between 1650 and 1540 cm^−1^, and bands near 1450 cm^−1^ and 1235 cm^−1^. These correspond to C=O stretching, N–H bending, and C–H vibrations of pyrrolidine rings, respectively. Their presence in both starch- and gluten-based films suggests that these absorptions mainly originate from gelatin, which was present in higher proportion in the formulations.

Wheat proteins contain sulfur-containing amino acids, and S–H stretching vibrations could theoretically appear between 2600 and 2550 cm^−1^, However, such bands were not detected. The S–H band is generally weak and may be masked by stronger absorptions, especially those associated with carboxyl groups. In addition, hydrogen bonding involving O–H and N–H groups is stronger than that involving S–H, which may further reduce the detectability of these vibrations [64].

The addition of 2% (*w*/*v*) potassium sorbate to GG non-irradiated films resulted in noticeable spectral changes, with fewer absorption bands compared with other spectra. This behavior may be attributed to the plasticizing effect of sorbate, which appears more pronounced in gluten-based films. This observation is consistent with the mechanical results presented in Table 3, where gluten-based films showed a greater reduction in tensile strength and increased elongation compared with starch-based films. The presence of potassium sorbate may reduce hydrogen bonding within the polymer network, decreasing vibrational intensity and leading to the disappearance of some bands.

Similar reductions in band intensity and spectral simplification after additive incorporation and irradiation have been associated with decreased intermolecular interactions and partial disruption of ordered structures [57].

Similar observations were reported by Vicentini [68], who investigated cassava starch and gluten films by infrared spectroscopy. Increasing gluten concentration altered band shape and intensity, decreasing peaks between 1200 and 900 cm^−1^ and increasing bands between 1700 and 1400 cm^−1^. According to that study, bands between 1650 and 1530 cm^−1^ correspond to the Amide I and Amide II bands of gluten proteins. The Amide I band reflects C=O stretching vibrations associated with protein secondary structure, whereas the Amide II band corresponds to combined N–H bending and C–N stretching vibrations of the peptide group –CO–NH.

Bands near 1650 and 1530 cm^−1^ were also observed in all the films. However, in composite systems containing gelatin, modified cassava starch or wheat gluten, potassium sorbate, and glycerol, the overlap of similar functional groups makes it difficult to attribute individual bands to specific components.

Overall, the FTIR results indicate that gamma irradiation at 2 kGy did not induce major chemical transformations in the polymer matrix but rather promoted subtle structural rearrangements, such as limited chain scission and modification of hydrogen bonding interactions. This behavior is consistent with the literature, which shows that low irradiation doses tend to cause mild degradation effects without significant alteration of the main chemical structure, whereas higher doses lead to pronounced bond cleavage and spectral intensity reduction [57,58,65]. Overall, FTIR analysis did not provide clear evidence that gamma irradiation caused chemical changes capable of significantly affecting antimicrobial diffusion in the films.

#### 3.1.6. Release of Potassium Sorbate from the Films

**Figure 7:**
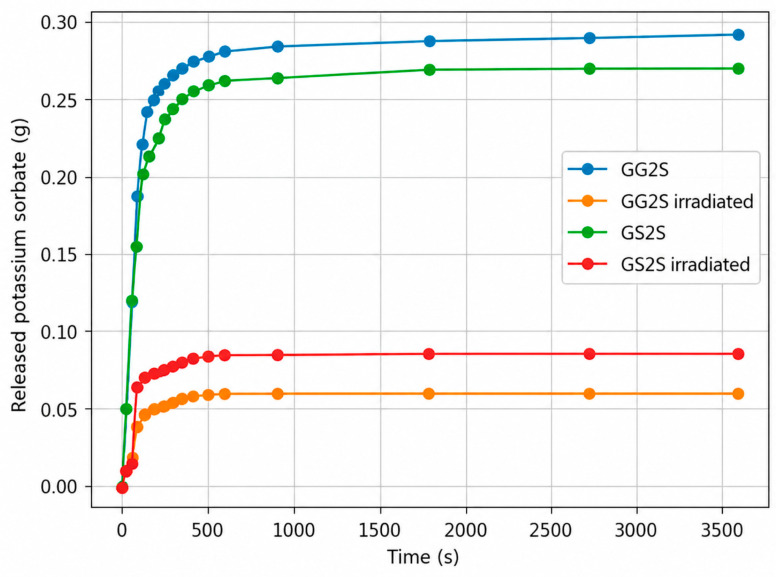
Kinetics of potassium sorbate release from GS2S and GG2S films as a function of time. GS = gelatin and starch; GG = gelatin and gluten; 2S = 2% potassium sorbate.

High-performance liquid chromatography (HPLC) was used to quantify the release of potassium sorbate from irradiated and non-irradiated films in order to evaluate whether differences in sorbate migration could explain the reduced antifungal activity observed in the halo inhibition assay (Figure 7). HPLC is widely used for the separation and quantitative determination of compounds in complex matrices due to its high sensitivity and analytical resolution [69]. Similar approaches have been applied to quantify sorbic acid in antimicrobial films and evaluate its migration into food systems [43,70].

Irradiated films released significantly lower amounts of potassium sorbate compared with the non-irradiated films for both formulations. In general, non-irradiated films released approximately five times more sorbate than irradiated films during the initial stages of the experiment.

The release profiles show a typical two-stage release behavior. An initial rapid release phase (burst release) was observed during the first minutes of the experiment, followed by a slower release stage approaching equilibrium (plateau). For non-irradiated films, sorbate release increased rapidly and began to stabilize after approximately 600 s, reaching equilibrium around 900 s. In contrast, irradiated films exhibited a slower release profile, with the reduction in sorbate release occurring around 150 s and equilibrium being reached at approximately 450 s. Quantitatively, GS2S films released approximately 68% more sorbate than GS2S irradiated films. Similarly, GG2S films released approximately 80% more sorbate than GG2S irradiated films.

These results indicate that gamma irradiation affected the diffusion behavior of potassium sorbate within the polymer matrix, reducing the release of the antimicrobial agent. When these results are considered together with the structural analyses performed in this study, a consistent interpretation emerges. FTIR analysis showed that the main functional groups associated with gelatin, starch, and gluten remained preserved after irradiation, indicating that no significant chemical modifications occurred in the polymer matrix. Likewise, SEM micrographs of both surface and cross-sections did not reveal major morphological differences between irradiated and non-irradiated films.

However, gamma irradiation may have induced subtle rearrangements within the polymer network, such as increased intermolecular interactions or partial crosslinking between polymer chains, which may reduce chain mobility and limit diffusion pathways for sorbate migration. In addition, the presence of glycerol as a plasticizer may influence the release behavior of the antimicrobial agent. In non-irradiated films, the plasticizing effect of glycerol likely increases polymer chain mobility and facilitates sorbate diffusion through the matrix. In contrast, irradiation may reduce this mobility by promoting stronger interactions between polymer chains, thereby restricting sorbate migration.

Among the different analyses performed to investigate the reduced antimicrobial effectiveness of irradiated films, the HPLC release analysis was the only method that provided quantitative evidence explaining this behavior. Although the release test was conducted in an aqueous medium, while the halo diffusion assay was performed in a solid culture medium with high water activity (aw ≈ 0.95), the results clearly demonstrated that irradiation significantly reduced potassium sorbate release from the films. Consequently, the reduced release of the antimicrobial compound likely explains the reduced fungal inhibition observed in the halo diffusion test.

Overall, these findings highlight an important trade-off in the development of irradiated active films. Although gamma irradiation may improve structural and mechanical properties of biopolymer-based films, it can also reduce the availability and release of the incorporated active compound. Therefore, the functionality of irradiated active packaging systems should not be evaluated only in terms of improved film properties, but also by considering the release behavior and antimicrobial effectiveness of the active agent. From an application perspective, these results indicate that optimizing release kinetics is essential to enhance antimicrobial performance. Strategies to improve sorbate release may include increasing plasticizer content to enhance chain mobility, adjusting polymer ratios to reduce matrix density, incorporating porogenic agents to create diffusion pathways, or designing multilayer systems to better control mass transfer. These approaches may help overcome the diffusion limitations observed in irradiated films and should be explored in future studies.

### 3.2. Food Application

#### 3.2.1. Application of Biofilms for Sliced Bread Packaging

Based on the results obtained for the active films four formulations were initially selected for application in packaging systems: (i) GS irradiated, (ii) GS2S irradiated, (iii) GG irradiated and (iv) GG2S irradiated. These formulations were selected considering their mechanical performance, barrier properties, and antimicrobial activity.

The films were applied to bread packaging using two approaches: (I) as primary packaging (standalone packaging) and (II) as interleaving materials between bread slices, which were subsequently placed in low-density polyethylene (LDPE) bags. In both cases, three slices were used per treatment. To enable their use as primary packaging, films with larger dimensions were required. Initially, films were produced using plates with a diameter of 12.5 cm, which limited their direct application as packaging. Therefore, a 40 × 40 cm acrylic plate was developed to produce larger films. The GG formulation showed good processability under these conditions, allowing the production of continuous and intact films using a casting volume of 180 mL, both with and without sorbate. In contrast, films based on GS, with and without sorbate, exhibited poor integrity during removal from the casting surface, leading to tearing and preventing their use at larger scale.

Although GS-based films exhibited higher mechanical strength, this characteristic, combined with the larger casting area, hindered their handling. Therefore, only GG irradiated and GG2S irradiated (2 kGy) films, were selected for packaging tests, considering their scalability and satisfactory antimicrobial performance.

#### 3.2.2. Weight Loss and Instrumental Texture Analysis of Bread Packaged with Films Applied as Primary Packaging

Weight loss results (Table 5) showed no significant changes (*p* ≤ 0.05) for samples packaged in low-density polyethylene (LDPE) throughout storage. In contrast, slices packaged with GG2S irradiated films exhibited a significant mass reduction (*p* ≤ 0.05), reaching approximately 16% at the end of storage. This behavior is attributed to the higher water vapor permeability of the biofilms, which promoted moisture loss. Consequently, a marked increase in firmness was observed, resulting in a texture comparable to toasted bread. No such changes were observed in LDPE-packaged samples.

Texture analysis (Table 4) confirmed these findings. While LDPE samples showed no significant variation (*p* ≤ 0.05), biofilm-packaged samples exhibited a substantial increase in firmness, from 85.52 ± 2.77 N (day 1) to 204.78 ± 2.95 N (day 7), corresponding to an increase of approximately 239%. From day 2 onward, firmness values were significantly higher than the control.

Notably, no fungal growth was observed in samples packaged with biofilms, whereas microbial growth occurred in LDPE samples from day 6. This effect is likely associated with reduced water activity.

Overall, despite their antimicrobial effect, the biofilms were unsuitable as standalone packaging due to excessive moisture loss and undesirable texture changes. These results indicate that the developed biofilms are not suitable as primary packaging materials, mainly due to their high-water vapor permeability and the resulting impact on product quality. From a practical perspective, this limitation highlights that biodegradable films with high hydrophilicity may not effectively replace conventional packaging systems when used alone. However, when applied as interleaving materials in combination with LDPE, the films showed a more balanced performance, suggesting their potential use in hybrid packaging systems. This indicates that such materials may be more suitable as complementary components rather than direct substitutes for conventional plastics, contributing to a gradual reduction in single-use packaging. Therefore, their practical application should consider the mode of use and interaction with the food system, particularly in terms of moisture transfer and texture preservation.

Based on the results obtained in this preliminary test, it was concluded that the bags developed with GG2S irradiated were not effective for packaging sliced bread. Therefore, subsequent experiments were conducted using stacked slices interleaved with active films and packaged in LDPE bags. Although no direct comparison was performed with commercially available biodegradable or conventional packaging materials, the present results provide relevant insight into the behavior of starch–protein-based films under controlled conditions. This study was designed to evaluate the intrinsic properties and functional response of these bio-based systems rather than to establish direct performance equivalence with commercial packaging. Future studies should include benchmarking against commercially available materials to better position these systems within the current packaging landscape.

#### 3.2.3. Application of the Biofilm to Stacked Bread Slices Interleaved with Active Films and Packed in LDPE Bags

GG irradiated and GG2S irradiated films (2 kGy) were applied between stacked bread slices and packaged in LDPE bags. The control consisted of slices packaged without films. Analyses were conducted over 7 days.

#### 3.2.4. Weight Loss and Texture (Firmness)

No significant differences (*p* ≤ 0.05) in weight loss were observed among treatments during storage (Table 5). The higher final weights in samples containing films are attributed to the additional mass of the films rather than to changes in bread moisture. Similar behavior has been reported for bread packaged with internal films, where weight variation is not significantly affected by active systems [44].

Firmness increased significantly over time for all treatments (Table 5), confirming the typical staling process of bread. This increase is mainly associated with starch retrogradation and water redistribution within the crumb [71,72]. From day 4 onwards, samples containing films showed significantly higher firmness compared to the control. This effect can be explained by the hydrophilic nature of gluten- and gelatin-based films, which promote water migration from the bread matrix to the film, intensifying crumb hardening. Similar moisture transfer effects have been described in protein-based edible films applied to bakery products [73].

#### 3.2.5. Moisture Content and Water Activity

Moisture values ranged from 28.74% to 33.60% (Table 5), consistent with typical bread composition [74]. A slight reduction in moisture was observed for all treatments, with lower values in samples containing films.

This behavior is attributed to moisture migration from the bread to the films, driven by differences in water content between phases. According to Labuza and Hyman [75], water transfer occurs until equilibrium is reached within the system. Despite this reduction, moisture loss alone does not fully explain bread staling, which is primarily governed by structural changes in starch [76].

Water activity decreased slightly during storage (0.97–0.92; Table 5), with more pronounced reductions in samples containing films. This trend is consistent with moisture migration to the films and the external environment.

However, aw values remained above 0.92 in all treatments, which is within the optimal range for microbial growth [77,78]. Similar results have been reported by Soares et al. [44], indicating that active films may influence water dynamics but are not sufficient alone to reduce microbial susceptibility.

The incorporation of GG irradiated films, especially with sorbate (GG2S), altered moisture distribution within the system, leading to increased firmness of bread slices during storage. These effects are mainly related to the hydrophilic nature of the films and their ability to absorb water, rather than to direct control of weight loss or water activity.

#### 3.2.6. Microbiological Analysis

Microbial growth is one of the main factors limiting the shelf life of bakery products. In this study, total yeast and mold counts, as well as total aerobic mesophilic counts (standard plate count), were performed to evaluate the microbiological stability of sliced bread during storage.

Yeast and mold enumeration was selected due to the susceptibility of bread to fungal spoilage. Analyses were conducted using Dichloran Glycerol Agar (DG18), which is specifically designed for xerophilic fungi and presents a water activity of 0.95, thereby reducing interference from bacteria and fast-growing molds [78]. Total aerobic mesophilic microorganisms were determined using Plate Count Agar (PCA), with a higher water activity (0.99), allowing the growth of both bacteria and fungi [78]. This analysis reflects raw material quality and processing, handling, and storage conditions, and is commonly used to estimate product shelf life [79]. Fungal growth may also favor the development of bacterial populations [80].

According to Brazilian regulations established by the Agência Nacional de Vigilância Sanitária (ANVISA) (RDC No. 331/2019 and IN No. 60/2019) [81,82], the maximum acceptable limit for yeasts and molds in bakery products is 4 log CFU/g. Based on this criterion, all treatments remained within acceptable limits at the initial storage time (day 1). On day 4, samples packaged in LDPE and GG2S irradiated film also complied with regulatory standards. However, by day 7, all treatments exceeded this threshold (Table 6), indicating microbiological spoilage and loss of product suitability.

**Figure A1:**
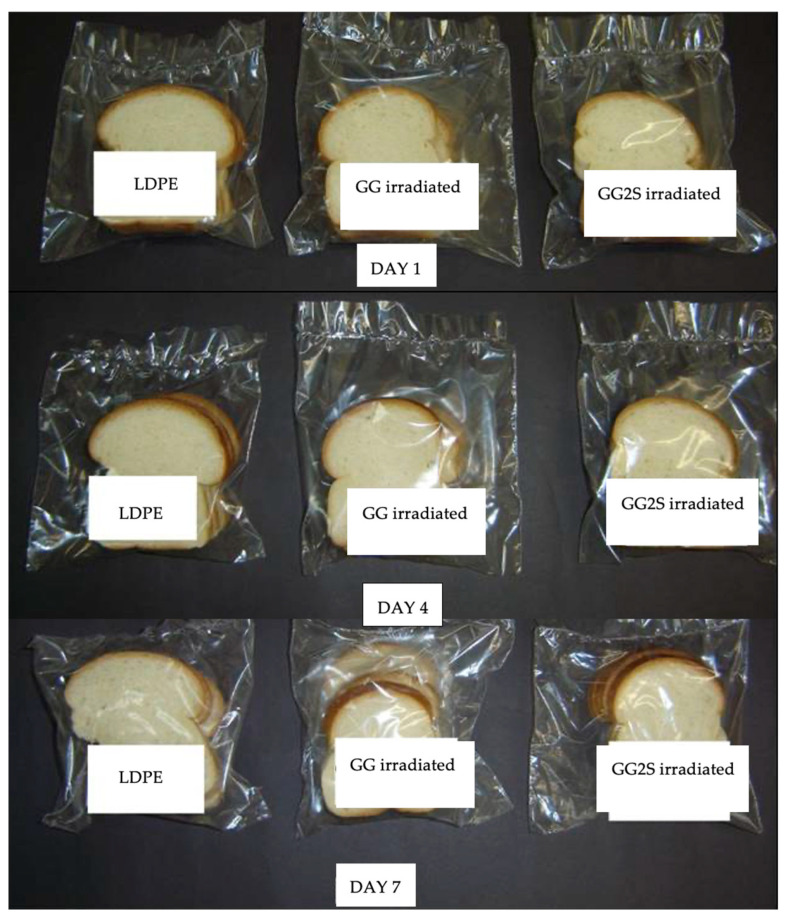
Visual appearance of sliced bread during storage. LDPE = low-density polyethylene GG = gelatin and gluten; 2S = 2% potassium sorbate.

A progressive increase in yeast and mold counts was observed during storage for all treatments (Table 6). No significant differences were detected between treatments on days 1 and 7 (*p* ≤ 0.05). However, on day 4, the GG2S treatment showed a significantly lower microbial count compared to the other treatments, indicating a temporary inhibitory effect. Despite this, fungal growth became visible after 9 days of storage for all samples (Figure A1), leading to early termination of the experiment.

For total aerobic mesophilic microorganisms, counts increased from approximately 1.10 log CFU/g on day 1 to values above 5.0 log CFU/g at the end of storage. A significant increase (*p* ≤ 0.05) was observed between days 1 and 4 for all treatments. From day 4 to day 7, this increase remained significant only for the GG2S irradiated treatment. Similarly to yeast and mold results, a significant difference between treatments was observed only on day 4, with lower counts for the sorbate-containing films.

Higher microbial counts in PCA compared to DG18 are attributed to the higher water activity of the medium, which supports the growth of both bacteria and fungi. In contrast, DG18 selectively favors fungal growth. The observed antimicrobial performance is consistent with previous studies. Soares et al. [44] reported that potassium sorbate incorporated into cellulose acetate films delayed microbial growth in bread, although no statistical analysis was provided. Similarly, Gunyunot et al. [83] demonstrated that potassium sorbate was the most effective preservative among tested compounds in bakery products with intermediate moisture and low pH.

It is also important to consider the role of irradiation (2 kGy) in the performance of the active films under real storage conditions. Ionizing radiation may induce chain scission in the polymeric matrix, reducing molecular weight and altering the three-dimensional network structure [56]. These modifications can increase free volume and hydrophilicity, potentially facilitating water uptake and enhancing the mobility of potassium sorbate. However, under practical conditions, such structural changes may also lead to a faster initial release followed by rapid depletion of the active compound, which can explain the transient antimicrobial effect observed on day 4.

Furthermore, the interaction between the irradiated film and the bread matrix likely influenced sorbate availability. Factors such as moisture migration from the bread, water activity, and partitioning of the antimicrobial compound between the film and the food system may have limited its effective concentration at the surface. Although irradiation may enhance diffusivity, the simultaneous increase in sorbate solubility and migration into the food matrix can reduce its localized antimicrobial action at the interface where microbial growth occurs.

Although GG2S irradiated films showed antimicrobial activity in agar diffusion assays, their performance in real food systems was limited. Under packaging conditions, microbial growth was similar or only slightly reduced compared to the control, indicating that the effectiveness of the active compound was reduced in the bread matrix. This behavior is consistent with HPLC results, which demonstrated that gamma irradiation reduced the release of potassium sorbate, thereby limiting its sustained availability and antimicrobial effectiveness under real storage conditions.

Overall, the antimicrobial performance of the developed films highlights the critical role of controlled release mechanisms in active packaging systems. Although potassium sorbate is a well-established antifungal agent, its effectiveness in the present study was limited by its reduced mobility and availability within the polymeric matrix. HPLC results demonstrated that gamma irradiation decreased the release of sorbate, which directly impacted its antimicrobial activity in both agar diffusion assays and real food systems. While irradiation may induce structural modifications such as chain scission and increased free volume, these changes did not translate into enhanced antimicrobial performance, likely due to restricted or non-sustained release of the active compound. This behavior explains the discrepancy between the intrinsic antimicrobial potential of sorbate and the modest inhibition observed, particularly under storage conditions where interactions with the food matrix, moisture migration, and partitioning effects further limit its availability. Therefore, the results emphasize that the success of antimicrobial films depends not only on the incorporation of active agents but also on the optimization of release kinetics, which must be tailored to ensure sustained and effective antimicrobial action throughout the product shelf life.

#### 3.2.7. Extraction and Quantification of Potassium Sorbate

According to Brazilian legislation [84] the maximum permitted level of sorbate in bakery products is 0.1 g per 100 g of product (0.1%). A significant increase (*p* ≤ 0.05) in sorbate content was observed over storage time, with values rising from 0.000026 ± 0.000005 g (day 1) to 0.000179 ± 0.000018 g (day 4) and reaching 0.002010 ± 0.00188 g (day 7) per 2 g of bread. This trend indicates a progressive release of sorbate from the GG2S irradiated active films into the bread matrix.

Considering that each package containing three slices weighs approximately 45 g, and that the legal limit corresponds to 0.045 g of sorbate per package, the extrapolated sorbate levels were 0.000585 g (day 1), 0.004027 g (day 4), and 0.045225 g (day 7). Therefore, by the seventh day, the amount of sorbate released approached the maximum limit established by legislation.

This behavior is consistent with diffusion-controlled release systems, in which the migration of active compounds is driven by concentration gradients and influenced by the hydrophilic nature of the polymeric matrix [73]. The gradual increase in sorbate concentration suggests continuous migration from the film to the food system over time.

Despite this release profile, the active films did not result in an effective extension of shelf life. Even at concentrations close to the regulatory limit, the antimicrobial effect was not sufficient to inhibit spoilage, likely due to the high-water activity of the product, which favors microbial growth.

## 4. Conclusions

Gamma irradiation and film composition played a decisive role in determining the functional performance of the developed active films. Among the evaluated conditions, 2 kGy was identified as the optimal irradiation dose, improving tensile strength in GG systems while reducing water vapor permeability without inducing excessive rigidity.

The incorporation of 2% potassium sorbate led to a tendency toward antimicrobial activity, with more pronounced effects against *Wallemia sebii*. However, no consistent statistically significant differences were observed between GG and GS films, indicating comparable antimicrobial performance across formulations.

Although no major structural changes were detected by SEM and FTIR analyses, HPLC results demonstrated that gamma irradiation significantly reduced sorbate release. This finding indicates that restricted diffusion, rather than chemical degradation, is the main factor limiting antimicrobial effectiveness in irradiated films. This behavior contrasts with studies reporting enhanced antimicrobial performance after irradiation at higher doses, highlighting that, at low doses, irradiation may instead impair functionality by limiting active compound mobility.

From an application perspective, GG films were the only formulations suitable for scale-up. However, when used as primary packaging, they promoted excessive moisture loss and undesirable increases in bread firmness. When applied as interleaving materials, the films altered moisture distribution and provided only temporary microbial inhibition, without significantly extending shelf life. Although sorbate migration increased over time and approached regulatory limits, it remained insufficient to effectively control spoilage under real conditions.

Overall, GG2S films represent the most promising system; however, their effectiveness is constrained by limited release kinetics and strong interactions with the food matrix.

These results demonstrate that irradiation-induced modifications in polymer structure do not necessarily translate into improved functional performance, particularly in hydrophilic biopolymer systems where mass transfer phenomena dominate.

More broadly, this study provides new insight into the structure–property–release relationship in irradiated starch–protein films, showing that the success of active biodegradable packaging depends not only on antimicrobial incorporation but on the precise control of release kinetics and moisture transport. These findings highlight a critical limitation in current bio-based systems and reinforce the need for designing hybrid or multi-layer strategies to achieve practical applicability in real food systems.

## Figures and Tables

**Table 1:** Effect of gamma irradiation dose on water vapor permeability and water solubility of selected films (25 °C).

Irradiated (kGy)	Films			
	GS	GS2S	GG	GG2S
Water vapor permeability (g·mm/m^2^·d·KPa)				
0	4.98 ± 0.18 ^c^	7.78 ± 0.75 ^ab^	4.46 ± 0.48 ^a^	7.07 ± 0.27 ^a^
2	4.00 ± 0.91 ^c^	4.87 ± 0.15 ^ab^	3.92 ± 0.33 ^a^	3.81 ± 0.18 ^b^
4	4.77 ± 0.38 ^c^	4.06 ± 0.55 ^b^	3.31 ± 0.70 ^a^	4.46 ± 0.46 ^b^
8	5.11 ± 0.19 ^bc^	5.34 ± 1.76 ^ab^	4.06 ± 0.78 ^a^	4.53 ± 0.47 ^b^
16	6.60 ± 0.52 ^ab^	5.15 ± 0.45 ^ab^	3.42 ± 0.11 ^a^	4.06 ± 0.39 ^b^
32	7.18 ± 0.18 ^a^	6.36 ± 0.11 ^a^	3.86 ± 0.25 ^a^	6.04 ± 0.05 ^a^
Solubility (%)				
0	38.63 ± 0.50 ^a^	40.49 ± 0.23 ^bc^	30.20 ± 0.18 ^bc^	40.73 ± 0.15 ^a^
2	35.66 ± 0.60 ^b^	40.21 ± 0.53 ^bc^	32.65 ± 0.96 ^a^	31.96 ± 1.84 ^bc^
4	30.70 ± 0.21 ^b^	32.94 ± 0.24 ^c^	29.04 ± 0.36 ^c^	25.09 ± 0.20 ^d^
8	38.23 ± 0.85 ^a^	38.42 ± 0.46 ^c^	31.03 ± 0.38 ^ab^	29.92 ± 0.70 ^c^
16	32.97 ± 0.89 ^b^	43.00 ± 0.66 ^a^	30.50 ± 0.10 ^bc^	25.81 ± 0.81 ^d^
32	36.79 ± 0.15 ^ab^	40.43 ± 0.61 ^b^	31.05 ± 0.67 ^ab^	34.93 ± 0.57 ^b^

Mean values ± standard deviation of replicates. Different letters in the same column indicate significant differences (*p* ≤ 0.05) according to Tukey’s test. GS = gelatin and starch; GG = gelatin and gluten; 2S = 2% potassium sorbate.

**Table 2:** Effect of gamma irradiation dose on tensile strength and elongation at break of selected films (25 °C).

Irradiated (kGy)	Films			
	GS	GS2S	GG	GG2S
Tensile strength (MPa)				
0	158.62 ± 2.94 ^a^	104.68 ± 7.56 ^a^	34.11 ± 4.59 ^d^	14.76 ± 1.52 ^c^
2	85.11 ± 4.28 ^d^	58.28 ± 5.31 ^cd^	68.05 ± 5.52 ^c^	61.89 ± 1.57 ^a^
4	85.92 ± 4.15 ^cd^	52.55 ± 5.66 ^d^	88.83 ± 2.53 ^b^	60.43 ± 3.72 ^ab^
8	94.50 ± 1.46 ^bc^	69.07 ± 7.64 ^c^	91.81 ± 6.57 ^b^	59.46 ± 1.60 ^ab^
16	92.24 ± 4.12 ^bcd^	82.84 ± 3.92 ^b^	107.04 ± 3.89 ^a^	54.39 ± 3.94 ^b^
32	95.62 ± 5.99 ^b^	85.57 ± 6.57 ^b^	105.41 ± 6.93 ^a^	53.63 ± 8.32 ^b^
Elongation at break (%)				
0	5.75 ± 0.58 ^a^	58.11 ± 7.46 ^a^	4.89 ± 0.43 ^a^	45.18 ± 5.48 ^a^
2	4.85 ± 1.54 ^a^	3.17 ± 0.33 ^b^	3.07 ± 0.93 ^a^	4.25 ± 0.64 ^b^
4	4.38 ± 0.89 ^a^	2.04 ± 0.32 ^b^	4.19 ± 2.31 ^a^	3.85 ± 0.62 ^b^
8	4.66 ± 1.68 ^a^	2.51 ± 0.98 ^b^	4.05 ± 0.72 ^a^	3.59 ± 1.10 ^b^
16	4.61 ± 1.80 ^a^	3.23 ± 0.50 ^b^	3.39 ± 1.24 ^a^	2.41 ± 0.38 ^b^
32	3.28 ± 1.54 ^a^	3.43 ± 0.73 ^b^	3.14 ± 0.78 ^a^	2.69 ± 1.07 ^b^

Note: Mean values ± standard deviation of replicates. Different letters in the same column indicate significant differences (*p* ≤ 0.05) according to Tukey’s test. GS = gelatin and starch; GG = gelatin and gluten; 2S = 2% potassium sorbate.

**Table 3:** Antimicrobial activity of irradiated (2 kGy) active films against different fungal strains evaluated by agar diffusion assay.

Fungal Strain	Film	CFU/mL	Inhibition Zone (mm)
*Aspergillus chevalieri*	GS irradiated	3.1 × 10^5^ ± 3.51 ^a^	0.00 ± 0.00 ^a^
	GS2S irradiated	2.2 × 10^5^ ± 5.29 ^a^	0.66 ± 0.57 ^a^
	GG irradiated	3.1 × 10^5^ ± 2.08 ^a^	0.00 ± 0.00 ^a^
	GG2S irradiated	1.9 × 10^5^ ± 6.11 ^a^	1.66 ± 1.15 ^a^
*Aspergillus montevidensis*	GS irradiated	1.8 × 10^6^ ± 0.57 ^a^	0.00 ± 0.00 ^b^
	GS2S irradiated	1.2 × 10^6^ ± 4.04 ^a^	1.33 ± 0.58 ^a^
	GG irradiated	1.5 × 10^6^ ± 4.04 ^a^	0.00 ± 0.00 ^b^
	GG2S irradiated	1.5 × 10^6^ ± 3.00 ^a^	1.33 ± 0.58 ^a^
*Wallemia sebii*	GS irradiated	3.7 × 10^6^ ± 6.11 ^a^	0.00 ± 0.00 ^b^
	GS2S irradiated	2.8 × 10^6^ ± 4.93 ^b^	1.33 ± 0.58 ^ab^
	GG irradiated	3.7 × 10^6^ ± 4.93 ^a^	0.00 ± 0.00 ^b^
	GG2S irradiated	2.5 × 10^6^ ± 5.29 ^c^	4.67 ± 2.52 ^a^

Notes: Values are expressed as mean ± standard deviation of replicates. Different letters indicate statistically significant differences (*p* ≤ 0.05) according to Tukey’s test. GS = gelatin and starch; GG = gelatin and gluten; 2S = 2% potassium sorbate.

**Table 4:** Weight loss and instrumental texture analysis of bread packaged with films applied as primary packaging.

Days	LDPE	GGS2 Irradiated	LDPE	GGS2 Irradiated
Weight Loss (g)			Firmness (N)	
1°	49.17 ± 0.21 ^aB^	75.34 ± 0.64 ^aA^	83.41 ± 2.03 ^aA^	85.52 ± 2.77 ^fA^
2°	49.30 ± 0.17 ^aB^	75.52 ± 0.76 ^bA^	83.53 ± 2.02 ^aB^	105.27 ± 3.17 ^eA^
3°	49.34 ± 0.26 ^aB^	69.30 ±0.59 ^cA^	83.02 ± 0.49 ^aB^	144.49 ± 3.93 ^dA^
4°	49.40 ± 0.09 ^aB^	66.73 ± 0.49 ^dA^	82.35 ± 0.99 ^aB^	166.04 ± 4.28 ^cA^
5°	49.48 ± 0.39 ^aB^	65.57 ± 0.11 ^deA^	81.24 ± 0.48 ^aB^	192.71 ± 3.34 ^bA^
6°	48.59 ± 0.28 ^aB^	64.82 ± 0.10 ^eA^	ND	169.75 ± 4.60 ^b^
7°	48.60 ± 0.38 ^aB^	63.25 ± 0.55 ^fA^	ND	85.52 ± 2.77 ^fA^

Note: Values are expressed as mean ± standard deviation of replicates. Different letters in the same row indicate significant differences (*p* ≤ 0.05) among means within the same sample, while different letters in the same column indicate significant differences (*p* ≤ 0.05) among means from different samples, according to Tukey’s test. GG2S irradiated = gelatin/gluten + 2% potassium sorbate (2 kGy); LDPE = low-density polyethylene; ND = not determined.

**Table 5:** Physicochemical properties of sliced bread during storage with LDPE and irradiated (2 kGy) packaging.

Day	Treatment	Weight Loss (g)	Firmness (g)	Moisture (%)	Water Activity (aw)
1	LDPE	45.94 ± 0.56 ^aB^	220.10 ± 5.56 ^cB^	33.60 ± 0.88 ^aA^	0.97 ± 0.00 ^aA^
	GG Irradiated	49.45 ± 0.68 ^aA^	228.88 ± 4.06 ^cA^	32.54 ± 0.65 ^aAB^	0.95 ± 0.01 ^ab^
	GG2S Irradiated	50.63 ± 0.91 ^aA^	225.91 ± 3.78 ^cAB^	30.60 ± 0.82 ^aB^	0.94 ± 0.00 ^aC^
4	LDPE	45.70 ± 0.55 ^aB^	433.58 ± 5.86 ^bC^	32.55 ± 0.71 ^aA^	0.96 ± 0.00 ^bA^
	GG Irradiated	49.16 ± 0.70 ^aA^	461.45 ± 3.44 ^bB^	30.59 ± 0.79 ^bAB^	0.94 ± 0.01 ^bA^
	GG2S Irradiated	50.35 ± 0.93 ^aA^	480.27 ± 5.69 ^bA^	29.07 ± 0.88 ^aB^	0.94 ± 0.00 ^aA^
7	LDPE	45.50 ± 0.57 ^aB^	626.80 ± 4.95 ^aC^	32.28 ± 0.30 ^aA^	0.94 ± 0.00 ^bA^
	GG Irradiated	48.76 ± 0.66 ^aA^	719.32 ± 4.85 ^aB^	30.27 ± 0.55 ^bB^	0.94 ± 0.00 ^bA^
	GG2S Irradiated	50.14 ± 0.93 ^aA^	726.97 ± 7.10 ^a^	28.74 ± 0.88 ^aB^	0.92 ± 0.00 ^bB^

Notes: Values are expressed as mean ± standard deviation. Different lowercase letters in the same column indicate significant differences (*p* ≤ 0.05) over storage time. Different uppercase letters in the same row indicate significant differences (*p* ≤ 0.05) among treatments (Tukey test). LDPE = low-density polyethylene; GG = gelatin and gluten; 2S = 2% potassium sorbate.

**Table 6:** Yeast and mold counts and total aerobic mesophilic counts of sliced bread during storage with LDPE and irradiated (2 kGy) packaging.

Days	Treatment	Yeasts and Molds (log CFU/g)	Total Aerobic Mesophiles (log CFU/g)
1	LDPE	<2.00 ± 0.00 ^cA^	1.10 ± 0.17 ^bA^
	GG Irradiated	<2.00 ± 0.00 ^cA^	1.10 ± 0.17 ^bA^
	GG2S Irradiated	<2.00 ± 0.00 ^bA^	<1.00 ± 0.00 ^cA^
4	LDPE	2.66 ± 0.73 ^bA^	4.86 ± 0.03 ^aB^
	GG Irradiated	3.97 ± 0.66 ^bA^	4.96 ± 0.27 ^aA^
	GG2S Irradiated	2.19 ± 0.36 ^bB^	3.93 ± 0.58 ^bB^
7	LDPE	5.72 ± 0.20 ^aA^	5.17 ± 0.82 ^aA^
	GG Irradiated	5.78 ± 0.43 ^aA^	5.16 ± 0.64 ^aA^
	GG2S Irradiated	4.96 ± 0.96 ^aA^	5.30 ± 0.30 ^aA^

Values are expressed as mean ± standard deviation of replicates. Different lowercase letters in the same column indicate significant differences (*p* ≤ 0.05) over storage time for the same treatment, while different uppercase letters in the same row indicate significant differences (*p* ≤ 0.05) among treatments, according to Tukey’s test. LDPE = low-density polyethylene GG = gelatin and gluten; 2S = 2% potassium sorbate.

## Data Availability

The data presented in this study are available on request from the corresponding author.

## Appendix A

**Table A1:** Standard sliced bread formulation.

Ingredients	Amount (% Relative to Flour)
Wheat flour	100
Water	58
Salt	2
Sugar	6
Instant dry yeast	1.3
Flour improver	0.3
Margarine	8
Whole milk powder	4
